# Deep Pyoderma Caused by *Serratia marcescens* in a Border Collie in China

**DOI:** 10.1002/vms3.70603

**Published:** 2025-10-31

**Authors:** Ran Wang, Ying Jiao, Yang Liu, Di Zhang

**Affiliations:** ^1^ College of Veterinary Medicine China Agricultural University Beijing China; ^2^ China Agricultural University Veterinary Teaching Hospital Beijing China

**Keywords:** bacterial resistance | canine | deep pyoderma | *Serratia marcescens*

## Abstract

This case report highlights *Serratia marcescens* as a causative agent of deep pyoderma in a Border Collie which presented with multiple erythematous lesions on the abdomen and back. The diagnosis of deep pyoderma was confirmed through clinical evaluation and bacterial identification. This case is the first reported instance of *S. marcescens* infection in a dog in China, and it was successfully managed with targeted antibiotic therapy.

## Graphical abstract

GRAPHICAL ABSTRACT

A 4‐year‐old Border Collie with deep pyoderma caused by *Serratia marcescens* exhibited erythematous patches over the abdomen and ruptured pustules scattered on the back. The dog was successfully treated with marbofloxacin at 2.7 mg/kg once daily.

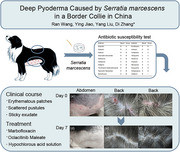

## 1 Case Information

1

A 4‐year‐old male castrated Border Collie, with vaccinations and deworming up‐to‐date, and undergoing annual routine physical examinations. The owner brought the dog in for a routine check‐up. During the abdominal ultrasound, the dog's abdominal hair was shaved, uncovering multiple erythematous lesions. The owner stated that the dog had similar skin symptoms in the same season 1 year ago, including scattered erythematous lesions on the back, along with significant dandruff, and occasional signs of ear scratching and paw licking. These signs resolved spontaneously within a few days without veterinary consultation or medical intervention. Over the following year, the dog remained in good health, with no recurrence of similar skin issues or other dermatologic abnormalities until the current episode. Two days prior to the visit, the dog had a bath at a grooming salon.

**FIGURE 1:**
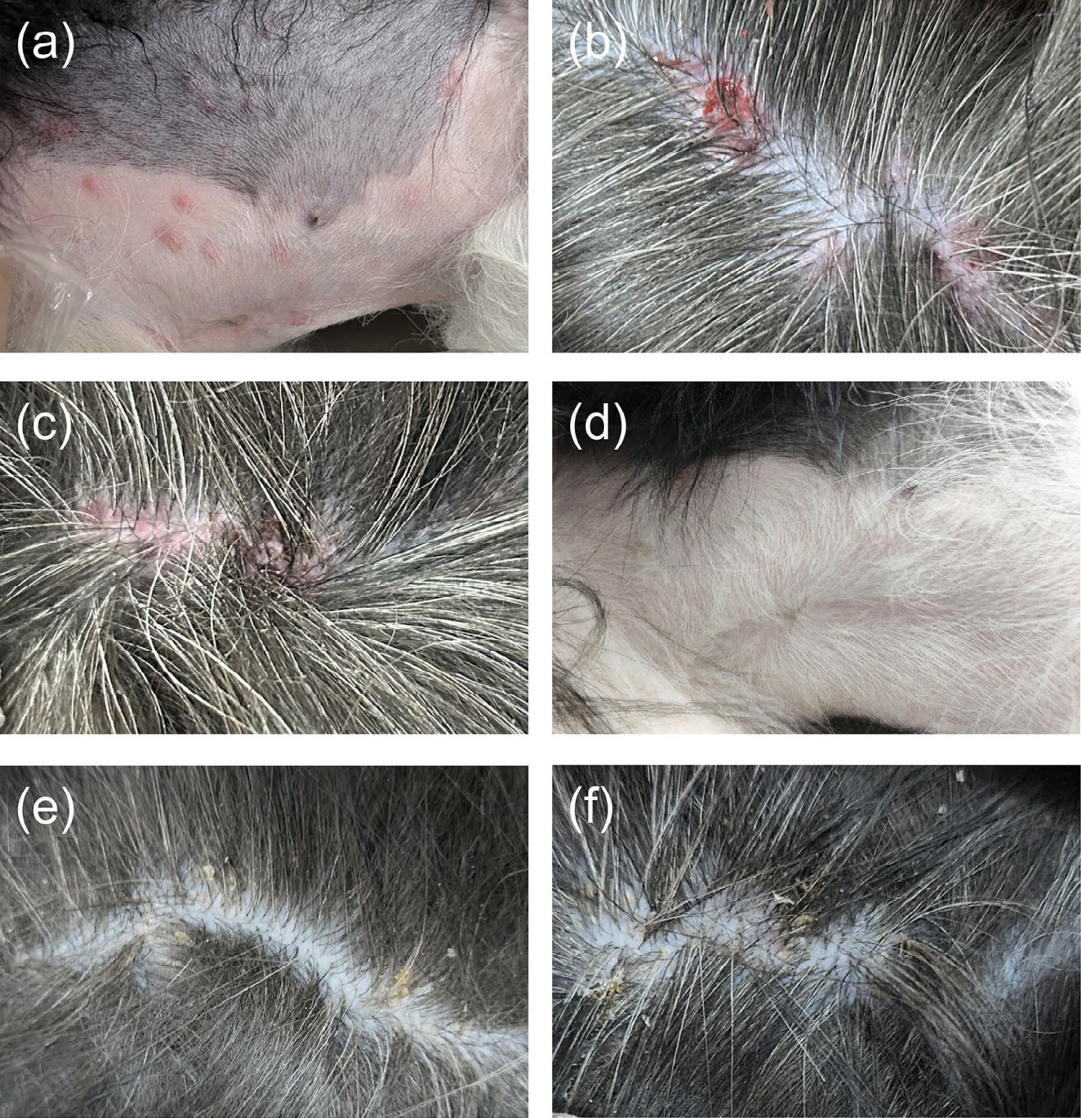
Affected skin of a 4‐year‐old neutered male Border Collie. (a) Multiple circular erythematous patches on abdomen. (b and c) Scattered pustules on the back. (d–f) No skin lesions observed after 7 days of treatment.

As referred to the dermatologist, the dog exhibited erythema over the abdominal skin, with multiple circular erythematous patches (Figure 1a), approximately 5 mm in diameter, slightly raised, which blanched with pressure. The back showed scattered pustules (Figure 1b,c), accompanied by sticky exudate, some of which had ruptured and crusted over. The skin temperature of the abdomen was elevated upon palpation, but no signs of pain or pruritus were noted.

There were no significant abnormalities in CBC and biochemical examination (Tables 1 and 2). Sterile disposable cotton swabs were used to collect skin samples from the lesion site. The samples were gently rolled onto glass slides, stained using the Diff‐Quick method and examined under a microscope. The cytological findings are summarized in Table 3.

**TABLE 1:** Results of complete blood count.

Item	Result	Reference range
RBC	5.95	(5.10–7.60) × 10^12/L
HCT	43.30	35.00%–52.00%
HGB	149.00	124.00–192.00 g/L
MCV	72.80↑	60.00–71.00 fL
MCH	25.00	22.00–26.00 pg
MCHC	344.00	320.00–380.00 g/L
RDW‐CV	12.70↓	13.20%–19.10%
RET‐He	21.40↓	22.30–29.60 pg
RET%	0.53	0.30%–2.40%
RET#	31.50	19.40–110.00 K/µL
NRBC%	0.20	0.00%–5.00%
NRBC#	0.03	
WBC	13.06	(5.60–18.40) × 10^9/L
NEUT#	10.09	(2.90–13.60) × 10^9/L
MONO#	1.10	(1.10–5.30) × 10^9/L
LYMPH#	1.83	(0.40–1.60) × 10^9/L
EO#	0.03↓	(0.10–3.10) × 10^9/L
BASO#	0.01	(0 ∼ 0.10) × 10^9/L
PLT	219.00	148.00–484.00 K/µL
MPV	7.90↓	9.10–12.70 fL
PCT	0.14	0.14%–0.46%

Abbreviations: BASO#, basophil count; EO#, eosinophil count; HCT, hematocrit; HGB, hemoglobin; LYMPH#, lymphocyte count; MCH, mean corpuscular hemoglobin; MCHC, mean corpuscular hemoglobin concentration; MCV, mean corpuscular volume; MONO#, monocyte count; MPV, mean platelet volume; NEUT#, neutrophil count; NRBC#, nucleated red blood cells count; NRBC%, nucleated red blood cells percentage; PCT, plateletcrit; PLT, platelets; RBC, red blood cells; RDW‐CV, red cell distribution width–coefficient of variation; RET#, reticulocyte count; RET%, reticulocyte percentage; RET‐He, reticulocyte hemoglobin equivalent; WBC, white blood cells.

**TABLE 2:** Results of biochemical examination.

Item	Result	Reference range
TBIL	0.15	0.00–0.80 mg/dL
TP	6.21	5.50–7.20 g/dL
ALB	4.00	3.20–4.10 g/dL
GLB	2.21	1.90–3.70 g/dL
A/G	1.81	0.90–1.90
GLU	104.64↑	68.00–104.00 mg/dL
FRU	352.58	266.00–381.00 µmol/L
ALT	41.60	17.00–95.00 U/L
AST	19.60	10.00–56.00 U/L
ALP	102.00	7.00–115.00 U/L
GGT	13.44↑	0.00–8.00 U/L
TBA	17.00	0.00–25.00 µmol/L
UREA	38.07	19.00–55.70 mg/dL
CREA	0.78	0.60–1.40 mg/dL
Ca	11.45	9.40–12.00 mg/dL
P	3.62	2.70–5.40 mg/dL
CHOL	263.66	136.00—392.00 mg/dL
TG	45.10	23.00–102.00 mg/dL
CK	49.30	0.00–314.00 U/L
K	4.23	4.10–5.40 mmol/L
Na	146.00	143.00–150.00 mmol/L
CL	114.00	106.00–114.00 mmol/L
cBNP	<500.00	0.00–900.00 pmol/L

Abbreviations: A/G, albumin/globulin ratio; ALB, albumin; ALP, alkaline phosphatase; ALT, alanine aminotransferase; AST, aspartate aminotransferase; Ca, calcium; cBNP, canine B‐type natriuretic peptide; CHOL, cholesterol; CK, creatine kinase; Cl, chloride; CREA, creatinine; FRU, fructosamine; GGT, gamma‐glutamyl transferase; GLB, globulin; GLU, glucose; K, potassium; Na, sodium; P, phosphorus; TBA, total bile acids; TBIL, total bilirubin; TG, triglycerides; TP, total protein; UREA, urea.

**TABLE 3:** Results of skin examination.

Item	Examination result
Bacteria	None observed
Fungi	None observed
Mites	None observed
Abnormal cells	Neutrophils: ++ Macrophages: +
Others	None observed

*Note*: The “+” indicates the presence of 10–20 cells per high‐power field (100× magnification). The “++” indicates the presence of 20–30 cells per high‐power field (100× magnification).

Given the abnormal characteristics of the skin lesions, skin samples were inoculated onto blood agar and MacConkey agar plates and cultured aerobically at 37°C for 24 h. On blood agar, numerous grey, slightly raised circular colonies were observed, exhibiting smooth, moist surfaces, regular edges and β‐haemolysis. On MacConkey agar, light pink, semi‐transparent, slightly raised circular colonies appeared, also with smooth, moist surfaces and regular edges. A single colony was isolated and identified as *Serratia marcescens* through MALDI‐TOF MS analysis. Following inoculation, a disk diffusion susceptibility test was performed, and the test report was issued according to the CLSI‐M100 and CLSI‐VET08 guidelines, as shown in Table 4. On the basis of the medical history, physical examination, skin examination and microbiological findings, a diagnosis of deep pyoderma caused by *S. marcescens* was confirmed.

**TABLE 4:** Results of the antibiotic susceptibility test.

Antibiotic	Result	Group	Antibiotic	Result	Group
Ampicillin	R	A	Enrofloxacin	S	B
Cefazolin	R	A	Amikacin	S	B
Cefoxitin	R	B	Ceftazidime	S	C
Amoxicillin–clavulanate	R	B	Tetracycline	S	C
Ceftriaxone	S	B	Florfenicol	S	C
Cefotaxime	S	B	Imipenem	S	C
Cefepime	S	B	Meropenem	S	C
Trimethoprim‐sulfamethoxazole	S	B	Doxycycline	S	O
Piperacillin–tazobactam	S	B	Cefoperazone	S	O
Marbofloxacin	S	B	Cefalexin	R	/

*Note*: A, Routine report drugs; B, drugs that can be used for routine testing but are reported selectively, when resistance is noted in similar drugs of Group A, they may be stopped; Group C, alternative or supplemental antimicrobial drugs; O, other drugs; “/”, no recommended grouping at this time.

Abbreviations: R, resistant; S, susceptible.

While awaiting the susceptibility test results, the dog was treated with amoxicillin–clavulanate (Synulox; Zoetis) at 18.0 mg/kg twice daily and oclacitinib maleate (Apoquel; Zoetis) at 0.6 mg/kg once daily. The affected skin was cleaned with a 2%–4% hypochlorous acid solution. Three days later, based on the aerobic culture and susceptibility test results, treatment was adjusted to include marbofloxacin (Marbocyl P; Vetoquinol) at 2.7 mg/kg once daily, while continuing oclacitinib maleate at 0.6 mg/kg once daily and the hypochlorous acid solution. Seven days later, the abdominal erythematous patches had resolved, the pustules on the back had crusted (Figure 1d), and dandruff was noted in the affected area (Figure 1e,f). Although the erythema had dissipated, occasional scratching persisted. After an additional 14 days of treatment, the dog was clinically cured, with all skin symptoms resolved and no further scratching behaviour observed. Five months after discontinuing the treatment, the dog remained in good condition with overall improvement and no recurrence of itching or similar skin symptoms.

## 2 Discussion

2

*S. marcescens* is a significant opportunistic pathogen commonly found in both natural environments and healthcare settings (Veraldi and Nazzaro 2016; Tavares‐Carreon et al. 2023). Infections caused by *S. marcescens* include fasciitis, endocarditis, respiratory infections, central nervous system infections and sepsis (Zivkovic Zaric et al. 2023). Iatrogenic transmission is a primary route of infection, as *S. marcescens* can persist on surgical instruments and in disinfectants, potentially causing outbreak infections (Coall et al. 2022; Kamali et al. 2024; Saralegui et al. 2020; Redondo‐Bravo et al. 2019). For instance, a 2020 report described an outbreak in a veterinary hospital, tracing the source to gauze pads impregnated with 1% chlorhexidine solution, which resulted in 32 cases of iatrogenic infection (Keck et al. 2020).

Reports of skin infections caused by *S. marcescens* are extremely rare. To date, only three cases of *S. marcescens* skin infection in dogs have been reported in the literature. In 2023, a veterinary hospital in Korea reported the first case of *S. marcescens* skin infection in a 12‐year‐old dog, which presented with persistent erythema and oedema on the ventral aspect of the tail, along with sanguineous fluid in the subcutaneous tissue (Koo et al. 2024). Subsequently, another veterinary hospital in Korea reported a second case involving purulent discharge on the nasal bridge (Park and Yoon 2024). Although the aforementioned cases and the present case all involve *S. marcescens* infection, there are notable differences in the dog's health status, infection site and symptoms. In the previous cases, the infection extended into deeper tissue layers and followed a more aggressive clinical course. This highlights the diversity in the clinical presentation of *S. marcescens* infections and the need for broader awareness of the varying manifestations and complexities of these infections in canines.

In this case, the dog had a bath at the grooming salon 2 days prior to the visit. Clinical examination revealed ruptured and scabbed erythematous lesions on the back, and scattered erythematous patches on the abdomen with localized warmth. No other abnormalities were noted. Given the dog's routine deworming, lack of recent medication or travel history and absence of oral mucosal abnormalities, a provisional diagnosis of deep pyoderma was made. Three days later, bacterial culture and susceptibility testing confirmed *S. marcescens* as the etiological agent.

Notably, microscopic examination of the initial skin smear revealed abundant inflammatory cells, but no visible bacteria. This finding was also observed in previous cases (Park and Yoon 2024), which may be related to the characteristic nature of *S. marcescens* skin infections or possibly due to a low local bacterial load or insufficient sampling depth. On the other hand, in this case, sampling was limited by crusted pustules on the back and residual ultrasound gel on the abdominal area, which may also have contributed to the negative cytological findings.

The underlying source of infection is likely related to grooming. Considering the timing of grooming, the location of the lesions and the clinical presentation, we strongly suspect that skin trauma during bathing, combined with subsequent infection, may have contributed to the development of deep pyoderma in this dog. A previous case of *S. marcescens* skin infection also had a history of bathing (Park and Yoon 2024). Traditionally, *S. marcescens* infections have been primarily associated with iatrogenic transmission; however, two of the four described cases suggest a possible link to bathing, highlighting a previously unrecognized susceptibility pathway. Although post‐grooming furunculosis is typically caused by *Pseudomonas aeruginosa*, there has been a report of a mixed infection involving both *S. marcescens* and *P. aeruginosa* (Cain and Mauldin 2015). This condition typically affects the dorsal neck and back, similar to the present case, although extensive lesions were also observed on the abdomen in this case.

The treatment strategy focused on infection control, pruritus management and skin cleansing.

*S. marcescens* is known to produce clinically significant AmpC β‐lactamase, which confers resistance to ampicillin, amoxicillin–clavulanate and first‐ and second‐generation cephalosporins, consistent with the findings in the present case report. Therefore, after performing a susceptibility test, the treatment was adjusted from the initial amoxicillin–clavulanate to marbofloxacin. According to the 2024 guidelines from the Infectious Diseases Society of America on managing infections caused by resistant Gram‐negative bacteria, most *S. marcescens* isolates remain susceptible to amikacin (Tamma et al. 2024), which is also consistent with the strain identified in this case and can serve as a reference for *S. marcescens* infections.

Pruritus was managed with oclacitinib maleate to prevent self‐trauma and preserve skin integrity. Topical treatments, including a 2%–4% hypochlorous acid solution and mousse, were applied to reduce foreign irritants and support the skin barrier. This comprehensive approach effectively controlled itching and prevented further complications.

No recurrence of the skin lesions has been observed to date. On the basis of the clinical signs observed 1 year prior and the breed's known predisposition, an allergic component cannot be entirely ruled out. However, aside from that earlier episode, there was no reported history of allergies. Given that the clinical diagnosis and confirmation of allergic conditions often involve a long and complex process, our initial diagnostic priority was to manage the active skin infection. The dog responded well to antimicrobial treatment. Therefore, we consider it unlikely that allergy played a significant role in the current episode. Nonetheless, we have advised the owner to continue monitoring the dog for any future signs suggestive of allergic disease.

Endocrinopathies are common underlying causes of dermatologic conditions in dogs. However, this patient was a relatively young Border Collie with no clinical abnormalities other than localized cutaneous lesions. Following consultation with an internal medicine specialist, further endocrine testing was deemed unnecessary at the time of diagnosis. Moreover, the dog showed complete resolution of clinical signs following a 21‐day course of treatment, with no recurrence observed over a 5‐month follow‐up period. Taken together with the absence of systemic signs, these findings suggest that an endocrine disorder was unlikely to have contributed to the current skin condition.

This case report has two main limitations. First, bacterial cultures of the shampoo and grooming brush were not performed, so the source of the *S. marcescens* infection could not be confirmed. Second, due to resource constraints, pathogen identification relied solely on MALDI‐TOF MS without the support of molecular detection or sequencing, which would have provided more comprehensive microbiological information. In clinical settings, molecular methods and sequencing are particularly advantageous for identifying rare or drug‐resistant bacterial species.

In conclusion, although skin infections caused by *S. marcescens* are relatively rare, they require careful attention in clinical practice. Prompt antimicrobial susceptibility testing and judicious antibiotic selection are essential when *S. marcescens* infection is suspected.

## Author Contributions

**Ran Wang**: writing – original draft preparation, writing – review and editing, visualization. **Di Zhang**: conceptualization, writing – review and editing. **Ying Jiao**: investigation, resources, visualization. **Yang Liu**: supervision, resources.

## Ethics Statement

The authors confirm that they have adhered to the journal's ethical guidelines, as set out on the journal's author guidelines page, and that written informed consent has been obtained from the patient's owner for the publication of this case report.

## Conflicts of Interest

The authors declare no conflicts of interest.

## Peer Review

The peer review history for this article is available at https://www.webofscience.com/api/gateway/wos/peer‐review/10.1002/vms3.70603.

## Data Availability

Data sharing not applicable—no new data generated.
